# Boosting Visible Light Photocatalysis: Se Rods Decorated with SnO_2_ Nanoparticles

**DOI:** 10.3390/ma18184300

**Published:** 2025-09-13

**Authors:** Stefania Mura, Pietro Rassu, Federico Fiori, Gabriele Masia, Sebastiano Garroni, Salvatore Marceddu, Ylenia Spissu, Luca Malfatti, Plinio Innocenzi

**Affiliations:** 1 Laboratory of Materials Science and Nanotechnology (LMNT), CR-INSTM, Department of Biomedical Sciences, University of Sassari, Viale S. Pietro 43/B, 07100 Sassari, Italyfederico.fiori@dottorandi.unipg.it (F.F.); luca.malfatti@uniss.it (L.M.); 2 Department of Chemistry, Physics, Mathematics and Natural Science, University of Sassari, Via Vienna 2, 07100 Sassari, Italy; g.masia12@studenti.uniss.it (G.M.); sgarroni@uniss.it (S.G.); 3 National Research Council of Italy (CNR), Institute of Sciences of Food Production (ISPA), Traversa La Crucca, 3-Regione Baldinca, Li Punti, 07100 Sassari, Italy; salvatore.marceddu@ispa.cnr.it (S.M.);

**Keywords:** tin oxide, selenium, photocatalysis, heterostructures

## Abstract

Se rods decorated with SnO_2_ nanoparticles have been synthesized via a facile hydrothermal approach to bridge the gap between ultraviolet-only and visible-only photocatalysis and to enhance reactive oxygen species generation under visible illumination. Structural and morphological analyses using X-ray diffraction and scanning electron microscopy with energy dispersive spectroscopy have confirmed the coexistence of cassiterite SnO_2_ particles intimately interfaced with trigonal selenium rods. Diffuse-reflectance spectroscopy revealed a long absorption tail extending into the 400–550 nm range. Under 450 nm sample illumination, the composite produced singlet oxygen in higher yields than either bare SnO_2_ or Se, as evidenced by the indocyanine green assay. The system alone does not produce free radicals, as shown by the terephthalic acid test; however, the addition of rhodamine B acts as an effective sensitizer, enabling hydroxyl radical generation. Photodegradation tests using rhodamine B have shown that the SnO_2_–Se system outperforms both its single components, Se and SnO_2_, as a catalyst. The synergistic interplay underscores the potential of SnO_2_–Se heterostructures in photochemical applications under visible light.

## 1. Introduction

The growing need for photocatalytic materials active in the visible region of the spectrum has led to the exploration of several inorganic nanosystems for this purpose. Traditionally, materials such as TiO_2_ (anatase) [1] and ZnO have dominated the field due to their high stability and low toxicity; however, they are only photoactive under UV irradiation because of their wide band gap (≈ 3.2–3.4 eV) [2]. To extend absorption to visible light, different strategies, including doping with heteroatoms (N, S, etc.) [3,4], dye sensitization [5], and the formation of heterostructures based on semiconductors with a smaller band gap [6], have been explored.

Among these approaches, mixed oxide heterostructures (TiO_2_–BiVO_4_ [7], ZnO–Cu_2_O, TiO_2−_ ZnO [8]) Refs. [9,10], sulfide- or telluride-based semiconductors (CdS [11,12] MoS_2_ [13] and CdTe [14]), and carbon-based compounds (g-C_3_N_4_ [15] and mixtures with oxides [16,17]) have shown a good compromise between visible absorption (≤2.5 eV) and charge separation. However, despite these advantages, many materials exhibit distinct drawbacks. For instance, one key challenge is rapid recombination: photogenerated lifetimes often remain below the microsecond range, thereby limiting charge transfer efficiency [18]. Additionally, material stability can represent a critical issue. For instance, some sulfide- [19] and telluride-based semiconductors [20] that are photoactive in the visible range tend to oxidize and release metal ions in aqueous environments [21]. Finally, while doping with transition metals or rare earths [22] improves visible absorption, it also raises the production costs and sustainability concerns [23].

SnO_2_–Se composites [24] represent an interesting combination between the wide-bandgap semiconducting nature of SnO_2_ and the unique electrical and optical properties of selenium-based materials [25]. The SnO_2_–Se combines two semiconducting materials with distinct local bonding geometries. These can be rationalized as follows. In SnO_2_, tin is in the +4 oxidation state and coordinated by six oxygen atoms, corresponding to an AX_6_ arrangement. This gives rise to an octahedral geometry and underpins the rutile-type crystal lattice of SnO_2_. In contrast, elemental selenium typically forms helical chains. Each Se atom is bonded to two neighbors and retains two lone pairs, leading to a bent geometry and explaining the angular structure of selenium chains. At the SnO_2_–Se interface, the rigid octahedral Sn–O framework is expected to interact with the bent Se units. The lone pairs on Se may potentially engage with Sn or O atoms at the surface. This heterostructure can enhance charge carrier separation, broaden light absorption, and tune electronic band alignments to improve performance across various devices [26]. Some examples have demonstrated the potential of SnO_2_-Se heterostructures [27,28], such as photocatalytic degradation of methylene blue [26,29] and high-sensitivity gas thermal sensing [30]; however, their properties and applications remain largely unexplored [31].

In this context, a SnO_2_–Se heterostructure, obtained by a simple and scalable hydrothermal method [32], would offer several advantages. It can extend absorption up to 550 nm thanks to the Se component, without resorting to toxic doping. An effective separation of charge carriers through a well-defined band alignment is also an advantage: electrons can move from Se to SnO_2_, while holes remain in Se, prolonging the lifetime of excited states.

The synthesis reported in the present work is straightforward: the material can be produced in a single step using a one-pot hydrothermal process at temperatures of 180 °C or lower, employing only commonly used reagents. This combination of properties makes the SnO_2_–Se system promising for applications in environmental decontamination and selective photocatalytic processes, exhibiting a removal of RhB [0.01 mg mL^−1^] of 36% in 120 min. This result offers a new perspective in the development of a new generation of catalytic composite systems that are photoactivated by visible light and are based on the formation of ad-hoc designed heterostructures.

## 2. Experimental

### 2.1. Materials and Methods

Selenium powder 99.99%, sodium borohydride (NaBH_4_), tin(II) chloride dihydrate (SnCl_2_ · 2H_2_O), rhodamine B, terephthalic acid, indocyanine green, and NaOH 35% were purchased from Sigma-Aldrich, USA. Milli Q water was used during the experiments. All reagents purchased were of analytical grade and used without further purification.

### 2.2. Synthesis of SnO_2_-Se Composites

**Scheme 1:**
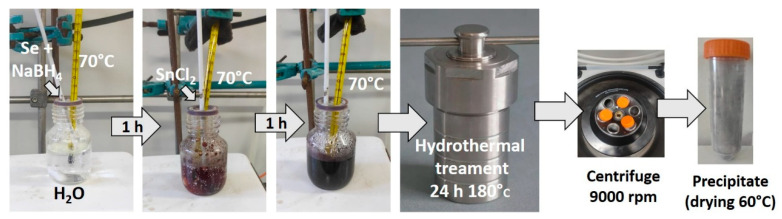
Synthesis steps, from the aqueous solution of Se + NaBH_4_ to the final dried powders.

The synthesis of the SnO_2_-Se composite was carried out hydrothermally by adding the components in solution in different steps. Initially, an aqueous suspension of selenium and sodium borohydride (NaBH_4_) was prepared. Selenium powder (Se) was used to form selenium anions by employing sodium borohydride as a reducing agent. In detail, 0.27 g of Se powder were mixed with 0.13 g of NaBH_4_ in 35 mL of mQ water and stirred homogeneously for 1 h at 70 °C. The initial solution was brown. After 1 h of stirring, 0.08 g SnCl_2_ · 2H_2_O were added and stirred for another hour. A dark green solution was initially obtained. As the reaction progresses, a black precipitate forms, accompanied by a yellow supernatant. Then, the solution was transferred to a 50 mL Teflon-lined autoclave for hydrothermal synthesis, heated at 180 °C in an oven in air, and left to react for 24 h. Finally, the solution was centrifuged at 9000 rpm for 20 min, and a black precipitate with a transparent supernatant was obtained, which was then discharged. The precipitate was finally put in an oven in air at 60 °C to dry until a gray-black solid was obtained (Scheme 1). Reference SnO_2_ was prepared by heating SnCl_2_ · 2H_2_O in an oven in air at 600 °C for 2 h.

### 2.3. Material Characterizations

UV-Vis spectra were recorded in the 200 to 800 nm range, on the powders pressed between two quartz slides of 1 × 1 cm in diffuse-reflectance and converted in absorbance, using an Agilent Cary series UV-Vis-NIR spectrophotometer.

Photoluminescence emission spectra were recorded using a spectrofluorometer (Horiba Jobin Yvon NanoLog) equipped with a 450 W xenon lamp as the excitation source. Fluorescence spectra of terephthalic acid were collected using a 2 nm slit, an excitation of 310 nm, and an emission of 315–600 nm.

Infrared spectra were obtained by using a Bruker Vertex 70 spectrophotometer in absorbance mode in the range of 4000–400 cm^−1^, with 4 cm^−1^ resolution and 128 scans, using KBr pellets as substrates. The baseline was fitted by a concave rubber band correction with OPUS™ 7.0 software, and data were analyzed by ORIGIN PRO™ software.

Raman spectra of SnO_2_-Se were collected in the 65–1555 cm^−1^ range with a 3–5 cm^−1^ resolution using a Senterra confocal Raman microscope (Bruker, 785 nm laser, 100 mW power, and 50× objective), irradiating the powders deposited on a Si wafer.

Structural characterization was performed by X-ray diffraction (XRD). The analyses were performed using a Smart Lab (Rigaku Corporation, Tokyo, Japan) diffractometer equipped with a Cu rotating anode (λ = 1.542 Å, 40 kV, 100 mA) in a Bragg–Brentano geometry. The XRD patterns were collected, setting a step size of 0.05° and a dwell time of 4 s per point, from 5° to 120°. The identification of the crystallographic phases was performed using the software Xpert Highscore v. 1.0b (Philips Analytical B.V., Almelo, the Netherlands).

The relative abundance of the crystallographic phases in the samples, as well as their microstructural parameters, was obtained by Rietveld refinement performed using the software MAUD v. 2.999 [33]. The structural data were compared to the crystallographic index files (CIFs) taken from the Crystallographic Open Database (COD) [34].

For SEM analysis, the samples were coated with carbon or gold in an Agar Auto Carbon Coater B7367A. The acquisition images and element analysis and mappings by energy dispersive spectroscopy (EDS) were conducted with a scanning electron microscope Zeiss EVO LS10 equipped with an Oxford INCAx-ACT EDS detector. The mean size of the particles was estimated by averaging at least 20 single measures from 5 different acquired images. The SEM was used in high vacuum mode, and the EDS analysis and element mappings were acquired using the AZtec software v 6.1 HF4.

### 2.4. Indocyanine Green Assay

Indocyanine green (ICG) was used to detect ^1^O_2_ [35]. Irradiation was performed in a home-built reactor using a 75 W blue LED strip (5 m long, STRIP COB HC264075 15 W/MT 24 V IP20 Blue) with λ = 450 nm. The strip was fixed to the surface of an aluminium can with glue and mounted on top of a magnetic stirrer. The temperature inside the photoreactor was kept at 25 °C with the aid of multiple fans and a thermometer. The ICG stock solution was prepared by dissolving 1 mg ICG in 10 mL mQ water. From this stock solution, 100 μL were taken and diluted to 2 mL with mQ water and used as a reference sample. Two mg of SnO_2_-Se, Se, and SnO_2_ powders were mixed with 100 μL ICG (stock) and diluted to 2 mL with mQ water. These two samples were irradiated with the previous LED at 450 nm, and UV-Vis spectra were collected from 0 to 120 min. The same experiment was also carried out, adding 200 μL RhB to the previous solution with ICG and SnO_2_-Se powder, and diluted to 2 mL with mQ water.

### 2.5. Terephthalic Acid Assay

Terephthalic acid (TA) has been employed to detect hydroxyl radicals (OH**^·^**) for its property of reacting with these radicals to form 2-hydroxyterephthalic acid (HTA), which is fluorescent [36]. The measurement of this fluorescence enables the quantification of OH radical concentration. A stock solution was prepared by dissolving 20 mg TA/50 mL of mQ water. To facilitate the dissolution of TA, 6.25 µL NaOH 35% were added. One mL of this solution was added to 2 mg of powders (SnO_2_-Se, SnO_2,_ or Se), and 2 mL of water were added, for a total of 3 mL of solution. For the experiment with the dye, 1 mL of TA solution was added to 2 mg of SnO_2_-Se powder and 200 µL of RhB (0.1 mg mL^−1^), resulting in a total solution volume of 3 mL in water. As a reference, 200 µL RhB were dissolved in 3 mL of water, and for TA measurement, 1 mL TA was dissolved in 3 mL of water. PL spectra were measured at t = 0 (t_0_) and after irradiation with a blue LED for 30 and 60 min.

### 2.6. Photodegradation Experiments of Rhodamine B

The samples were prepared by dissolving 200 µL of RhB (0.1 mg mL^−1^) in 2 mL of mQ water as a standard. The test samples were prepared as before, but with the addition of 2 mg of SnO_2_–Se, SnO_2_ and Se powder. The UV-Vis spectra were collected at t_0_ and after irradiation with LEDs at 30 min intervals for a total of 120 min. For the experiment, the blue LED was operated at its maximum irradiation power (100%, 75 W), and the cuvette was placed 10 cm from the reactor wall.

## 3. Results and Discussion

**Figure 1:**
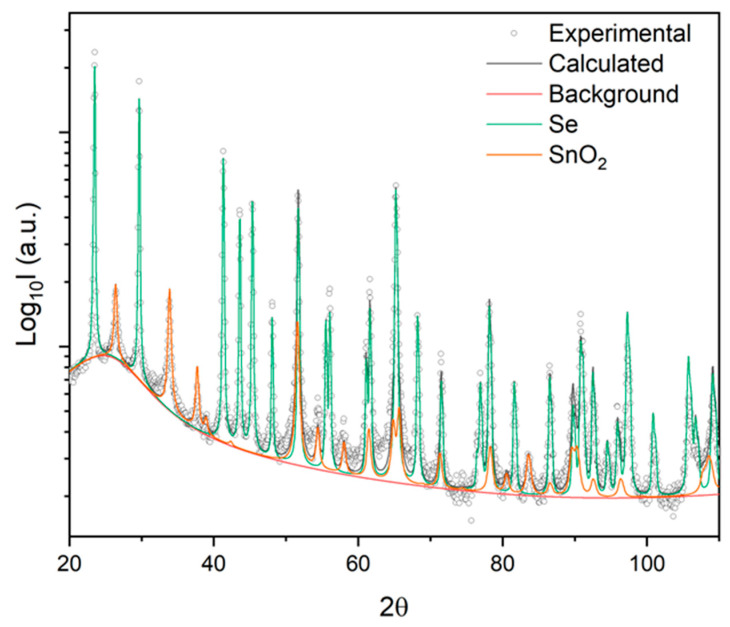
XRD patterns of SnO_2_–Se are shown as black hollow dots with a black line representing the Rietveld fit. The fit has been done using the experimental data of SnO_2_ cassiterite tetragonal rutile structure (orange line) and the trigonal Se (green line).

The formation of a composite, such as SnO_2_–Se, was initially studied using X-ray diffraction analysis to investigate the crystalline phases formed in the heterostructured material (Figure 1).

The analysis of the SnO_2_–Se powders shows the presence of two distinct crystalline phases, SnO_2_ cassiterite tetragonal rutile structure (JCPDS card n. 41-1445) and trigonal Se (JCPDS card n. 06-0362), the thermodynamically stable form of crystalline Se at room temperature. Interestingly, the formation of two phases is in contrast with what was observed by Reddy et al. [32], who obtained a single SnSe_2_ phase using a similar synthesis method. SnSe_2_ is a layered dichalcogenide with a hexagonal structure [37] with the main reflection peaks at ~14.4° (001), I_100_, and ~29° (002) I_60–70_. In our case, however, we have not employed an inert atmosphere, and a Se–SnO_2_ structure, rather than SnSe_2_, has formed because the atmosphere contains oxygen.

The XRD data well support the hypothesis of the formation of a Se–SnO_2_ nanostructured composite, consisting of ~91% trigonal Se and ~9% tetragonal SnO_2._ The average crystallite dimensions have been calculated by the Rietveld method, SnO_2_ = 29.0 and Se = 148.8 nm. The eventual presence of SnSe_2_ can be ruled out because no corresponding diffraction signals have been detected.

**Figure 2:**
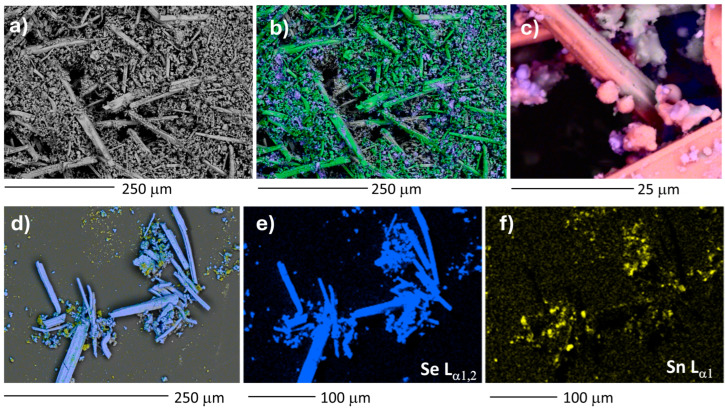
(**a**) SEM image of the SnO_2_–Se composite. (**b**) Energy dispersive spectroscopy (EDS) map of image in a, showing the distribution of Sn (in violet) and Se (in green) elements. (**c**) SEM image with the detail of the composite and the distribution of Sn (blue) and Se (red) elements. (**d**) SEM image showing the formation of two distinct structures, rod-like crystals and spherical particles. (**e**) Distribution of Se in image d and (**f**) distribution of Sn in image d.

The XRD data indicate the formation of heterostructures composed of crystalline Se and SnO_2_. To get a direct insight into the material morphology, we have analyzed the samples by SEM and EDS (Figure 2).

The SEM and EDS analysis shows that the SnO_2_–Se is formed by two distinct structures with well-defined shapes (Figure 2a). The first one consists of elongated rods measuring a few hundred microns. Together with these rods, spherical particles of sub-micron size are also observed. EDS analysis has enabled the distinction between the two structures: the more elongated ones are composed of selenium crystals, while the particles are composed of tin dioxide (Figure 2b–f). By comparing the XRD and SEM results, we conclude that both the Se and SnO_2_ structures exhibit a polycrystalline nature.

**Figure 3:**
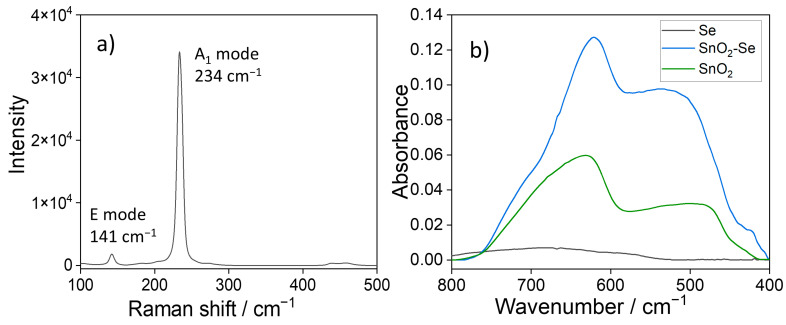
(**a**) Raman spectra and (**b**) FTIR absorption spectra of the SnO_2_–Se powder. The infrared spectra of Se and SnO_2_ are reported as a reference.

To investigate the structure of the nanostructured composite, we have also employed Raman and FTIR spectroscopy as supporting evidence. Figure 3a shows the Raman spectrum of the SnO_2_–Se sample in the 100–500 cm^−1^ interval; no signals have been observed beyond this range. The spectrum exhibits two intense bands peaking at 141 and 234 cm^−1^. The first band corresponds to the transverse optical phonon mode (E mode) [38]. The second band, observed at higher wavenumbers, is characteristic of trigonal selenium (t-Se) and is assigned to the stretching vibration of helical selenium chains (A_1_ mode). The weak band around 459 cm^−1^ is, instead, attributed to the first overtone of t-Se A_1_ mode [39].

We have used infrared spectroscopy as a complementary technique to Raman spectroscopy. Figure 3b shows the FTIR absorption spectra in the 800–400 cm^−1^ range of the as-synthesized SnO_2_–Se powder with the Se and SnO_2_ used as a reference. (Full spectra are reported in SI1.) The SnO_2_–Se infrared spectrum shows two broad absorption bands peaking at 620 and 534 cm^−1^ [40,41]. The SnO_2_ sample shows a similar spectrum, while Se does not absorb infrared light in the same range. The FTIR spectrum of cassiterite SnO_2_ is dominated by a broad E_u_-derived antisymmetric stretch peaking at 629 cm^−1^ and a weaker A_2u_ bending around ~547–610 cm^−1^ [42]. The same features are observed in the SnO_2_–Se sample at 620 and 534 cm^−1^, confirming the formation of the SnO_2_ phase [43]. The SnO_2_–Se spectra suggest the presence of a nanosized fraction of SnO_2_ particles. In fact, as the particle size decreases, the lower-frequency component (∼534 cm^−1^) grows in relative intensity, and the high-frequency stretch peak (∼620 cm^−1^) can shift slightly toward lower wavenumbers (red-shift) due to phonon confinement and increased lattice strain [44,45].

**Figure 4:**
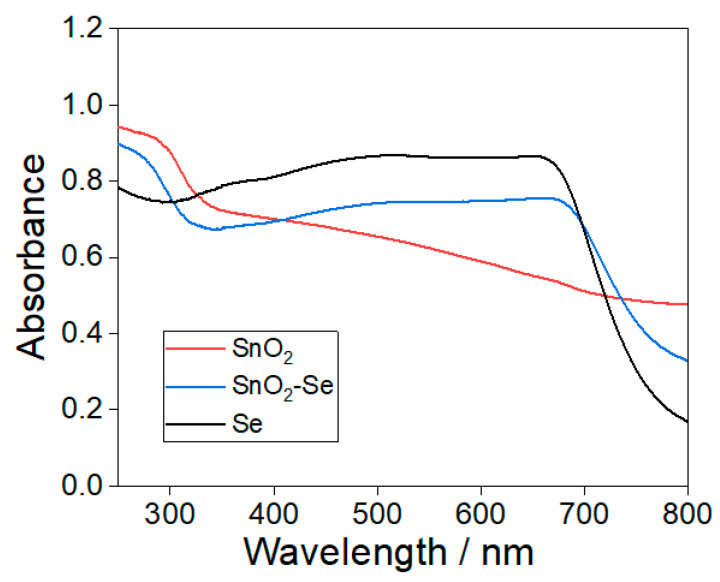
Diffuse reflectance UV-Vis absorption spectra of SnO_2_, Se, and SnO_2_–Se.

Figure 4 shows the diffuse reflectance UV-Vis absorption spectra of Se–SnO_2_ and reference Se and SnO_2_. The Se spectrum is characterized by an absorption onset around 690 nm, corresponding to an indirect band gap of 1.71 eV (calculated by Tauc plot). SnO_2_, on the other hand, is a direct band gap semiconductor and has a sharp absorption edge that typically starts around 330–350 nm, corresponding to a band gap of 3.73 eV. Interestingly, in the Se–SnO_2_ composite, a longer tail extending from the Se band edge up the SnO_2_ visible region, due to interfacial and defect states, is observed [46]. Figure 4 illustrates that the SnO_2_–Se system exhibits enhanced absorption in the visible region, which increases at longer wavelengths, thereby improving sensitivity in the red region. In general, the particle size of a semiconductor influences light absorption. An increase in surface area leads to more surface defects, which create localized states within the bandgap and promote defect-related sub-bandgap absorption. In our system, however, the Se rods decorated with SnO_2_ nanoparticles are on the micron scale, suggesting that the extended absorption originates from Se–SnO_2_ interfaces rather than size effects.

**Figure 5:**
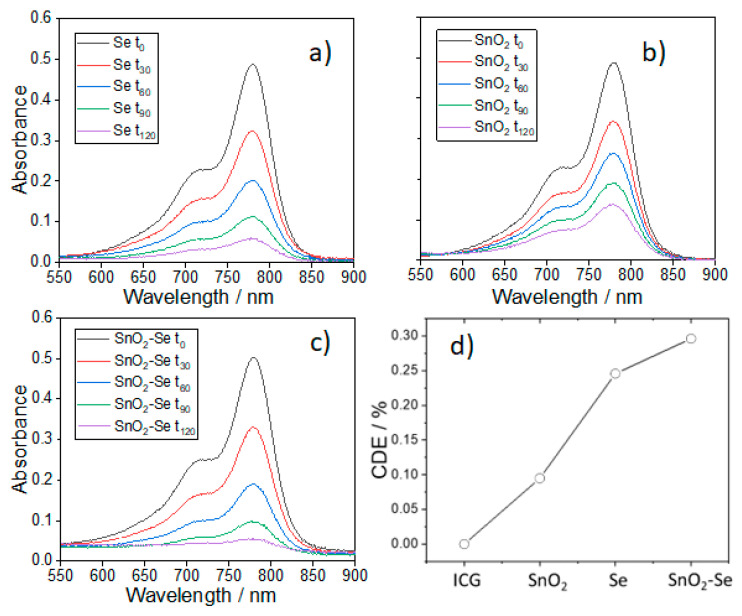
ROS emission test by indocyanine (IGC) assay. Optical absorption spectra of an aqueous solution of indocyanine and (**a**) Se; (**b**) SnO_2_ and (**c**) SnO_2_–Se samples upon exposition to blue light (λ = 450 nm) for different times, up to 120 min (t_120_). (**d**) Comparative degradation efficiency (CDE).

To assess the production of radicals or ROS by SnO_2_–Se when illuminated by visible light, two tests have been performed. The first one is the green indocyanine (IGC) assay (Figure 5). ICG, which has an absorption peak around 780 nm in aqueous solution, is stable under visible-light irradiation in the absence of ^1^O_2_ but undergoes oxidative cleavage of its polymethine chain in the presence of singlet oxygen [47]. The net effect is a decrease in the ICG absorbance at ~780 nm that is proportional to the cumulative amount of ^1^O_2_ generated in the system [48].

Figure 5a–c show the effect of the exposure to blue light (λ = 450 nm) of an aqueous solution containing ICG, the reference samples Se and SnO_2_, and the SnO_2_–Se. The test shows a time-dependent reduction in the absorbance of the 780 nm band for all samples, indicating the formation of singlet oxygen upon exposure to visible light [49]. Figure 5d displays the comparative degradation efficiency (CDE) calculated by the following formula [50]:

CDE (%) = 1 − [A_0 (ICG)_ − A_120 (ICG)_]/[A_0 (sample)_ − A_120 (sample)_] (1)

The ICG experiment indicates that while both SnO_2_ and Se are still capable of generating singlet oxygen upon illumination with blue light, the SnO_2_–Se system enhances the emission.

**Figure 6:**
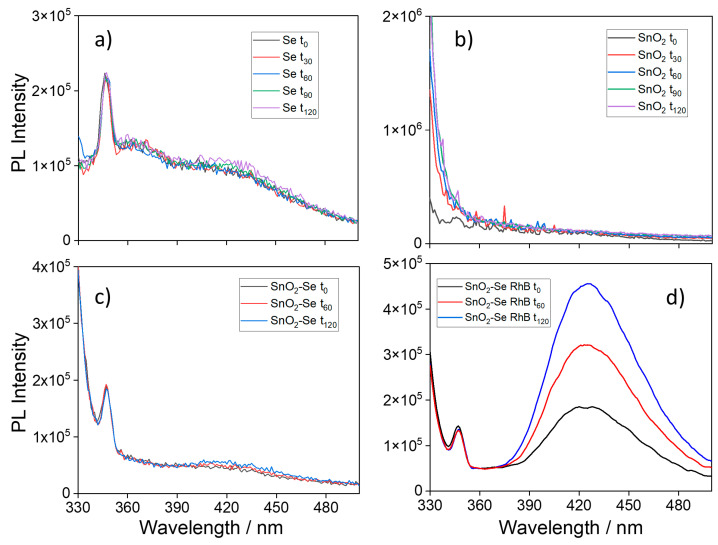
Photoluminescence emission spectra of terephthalic acid measured in solutions containing the different samples and exposed to 450 nm radiation for different times (from 0 (t_0_) to 120 (t_120_) minutes). (**a**) Se; (**b**) SnO_2_; (**c**) SnO_2_–Se; and (**d**) SnO_2_–Se powder with the addition of RhB.

The other assay is based on terephthalic acid (TA) [51], which is a selective chemical probe for hydroxyl radicals (^•^OH) (Figure 6). In aqueous solution, TA itself is essentially non-absorbing and non-fluorescent in the near-UV/visible region. When ^•^OH attacks TA, it hydroxylates the aromatic ring to give 2-hydroxyterephthalic acid (2-HTPA), which exhibits a characteristic fluorescence at ≈425 nm that can be used for the test. The test has been performed using Se (Figure 6a) and SnO_2_ (Figure 6b) samples as references, and no formation of radicals has been observed. Exposure to visible light (450 nm) does not produce any hydroxyl radicals, as no significant changes in the fluorescence band at 425 nm are detected either for the SnO_2_–Se system (Figure 6c). The two tests, ICG and TA assays, demonstrate that the SnO_2_-decorated Se rods, upon excitation by visible light, generate singlet oxygen but not hydroxyl radicals.

Interestingly, the same test repeated by adding RhB to the Se, SnO_2_ and SnO_2_–Se aqueous suspensions has shown different results. In Se and SnO_2_ samples, no differences have been observed, while the SnO_2_–Se sample shows a significant change. The hydroxyl radical (^•^OH) detection test shows, in fact, an increased fluorescence intensity of the band assigned to the formation of 2-HTPA (around 425 nm), indicating an enhanced production of hydroxyl radicals (Figure 6d).

In the terephthalic acid assay, only the ^•^OH (hydroxyl) radicals generated under illumination are detected. Neither bare Se nor bare SnO_2_ produces a measurable ^•^OH signal because SnO_2_ is a wide-band-gap semiconductor (≈ 3.6 eV) that mainly absorbs UV light (< 350 nm). Under typical visible-light or near-UV illumination used in many radical tests, SnO_2_ is not photoexcited enough to drive water oxidation to ^•^OH. On the other hand, crystalline Se absorbs more in the visible spectrum; however, as a zero-band-gap or very small-band-gap “semimetal,” photogenerated carriers recombine very quickly. Crystalline selenium efficiently produces singlet oxygen (see Figure 5a) because the absorbed photon energy is almost exclusively involved in the generation of triplet excitons. These in turn transfer energy to O_2_ to form singlet oxygen with high quantum yield. In the absence of a photosensitizer, the energy transfer mechanism to convert O_2_ into ^1^O_2_ is much more efficient than the multistep redox sequences required to produce ^•^OH species. Finally, in crystalline selenium, there is no built-in electric field or heterojunction to pull electrons and holes apart, so almost no oxidative chemistry (^•^OH formation) succeeds before recombination. In both bare materials, any electrons and holes that form upon light absorption recombine on a sub-nanosecond timescale, while an efficient spatial separation of charges is necessary to allow holes to oxidize surface water (H_2_O → ^•^OH + H^+^ + e^−^).

**Scheme 2:**
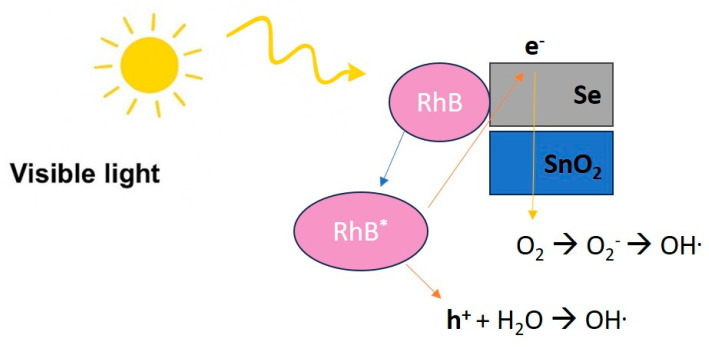
Photosensitization mechanism in the SnO_2_–-Se + rhodamine B (RhB) system.

On the other hand, rhodamine B strongly absorbs visible light, exhibiting a broad absorption band peaking around 550 nm. When the SnO_2_–Se–RhB system is irradiated with visible light, RhB is excited (RhB → RhB*) and can inject an electron into the Se valence or conduction band (depending on alignment), which then cascades into SnO_2_ [52]. The extra injected electron leaves behind an RhB^+•^ radical cation; the photogenerated hole on RhB (or in Se) is highly oxidizing and can oxidize surface H_2_O or OH^−^ to ^•^OH. Meanwhile, the transferred electron in SnO_2_ can reduce O_2_ to superoxide, which may further form ^•^OH via secondary pathways. The SnO_2_–Se + RhB system benefits from dye sensitization, efficient heterojunction charge separation, and appropriate surface reaction sites, enabling the holes (and/or superoxide intermediates) to oxidize water into detectable ^•^OH radicals [53]. The photosensitization mechanism of the SnO_2_–Se + RhB system is sketched in Scheme 2.

**Figure 7:**
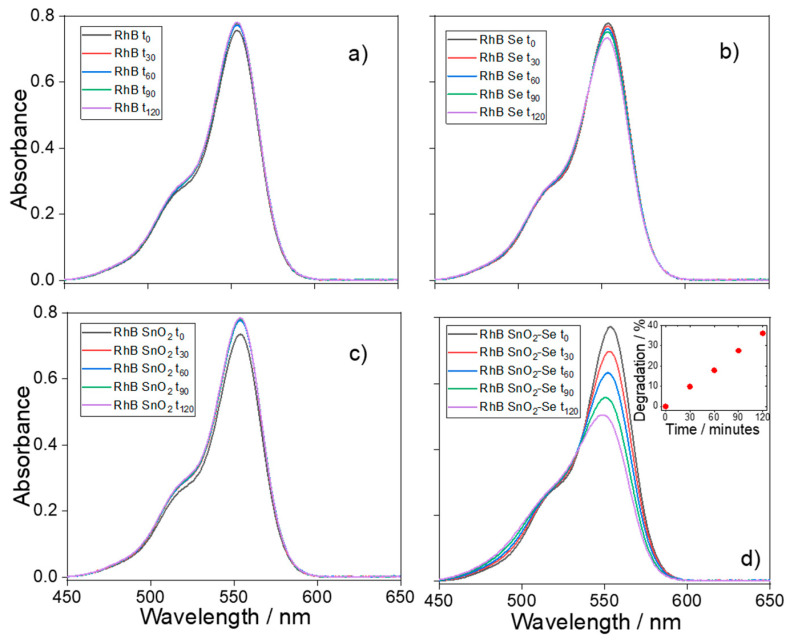
UV-Vis absorption spectra of RhB illuminated by blue light (450 nm) for different times, up to 120 min (t_120_). (**a**) RhB; (**b**) RhB + Se; (**c**) RhB + SnO_2_; and (**d**) RhB + SnO_2_–Se (the RhB degradation percentage with time is reported as inset).

We then performed another experiment using only RhB as the test molecule. RhB does not absorb light under 450 nm, and under illumination by blue light, it does not degrade (Figure 7a). The addition of Se (Figure 7b) or SnO_2_ (Figure 7c) to the aqueous solution produces only a slight reduction in the absorption maxima. If, instead, SnO_2_–Se (Figure 7d) is added to the RhB solution, a 36% absorbance reduction is observed.

The result confirms that the SnO_2_–Se system plays a synergistic role. Under 450 nm illumination, only materials whose absorption edge extends into the blue can be photoexcited, and only systems that can both separate those photo-carriers and channel them into redox chemistry will drive dye degradation. This explains why the SnO_2_-decorated Se rods work well when either component alone fails. The SnO_2_–Se heterostructure combines visible-light harvesting, efficient charge separation, and parallel ROS-forming pathways, enabling rapid RhB degradation under 450 nm light that neither bare SnO_2_ nor bare Se can achieve. The mechanism of photodegradation can be summarized with the following steps:

1. Absorption and excitation:

SnO_2_ (Se) + Photon (450nm) → SnO_2_ (Se)* (excited state)

2. Charge separation:

SnO_2_* → e^−^ + h^+^ (electron and hole generation)

3. Electron transfer to Se (ROS formation):

e^−^ (in Se) + O_2_ → O_2_^●−^ (superoxide anion formation)

h+ (in SnO_2_) + H_2_O → OH^●^ + H^+^ (hydroxyl radical formation)

4. Dye degradation via ROS:

RhB + OH^●^ + O_2_^●−^ → degradation products

The rhodamine B test is also an indication of the photodegradation capability of the SnO_2_–Se heterostructure. The graphical representation of the RhB degradation with time (inset of Figure 7d) reveals that after 120 min, the SnO_2_–Se allows achieving a 36% removal of RhB [0.01 mg mL^−1^].

## 4. Conclusions

In this study, we have demonstrated that SnO_2_-decorated Se rods synthesized via a simple one-pot hydrothermal method exhibit enhanced visible-light photocatalytic performance compared to their individual components. Under 450 nm irradiation, the composite generates singlet oxygen (^1^O_2_) more efficiently than the individual components, as confirmed by the indocyanine green assay. Photodegradation of rhodamine B under visible light outpaces that of bare SnO_2_ and Se. The absence of toxic dopants or noble metals, together with mild hydrothermal synthesis conditions, highlights the scalability and environmental friendliness of SnO_2_–Se composites compared to existing visible-light photocatalysts. Future work should focus on in-depth spectroscopic studies (e.g., time-resolved photoluminescence, transient absorption) to directly quantify interfacial charge dynamics. Moreover, extending this heterostructure concept to other chalcogen–oxide combinations is a feasible route for visible-light-active photocatalysts in environmental and synthetic photochemistry.

## Data Availability

The original contributions presented in this study are included in the article material. Further inquiries can be directed to the corresponding authors.
